# Kinetic study on the inhibition of xanthine oxidase by acylated derivatives of flavonoids synthesised enzymatically

**DOI:** 10.1080/14756366.2017.1347165

**Published:** 2017-07-18

**Authors:** Maria Elisa Melo Branco de Araújo, Yollanda Edwirges Moreira Franco, Thiago Grando Alberto, Marcia Cristina Fernandes Messias, Camila Wielewski Leme, Alexandra Christine Helena Frankland Sawaya, Patricia de Oliveira Carvalho

**Affiliations:** a Laboratory of Multidisciplinary Research, São Francisco University, Bragança Paulista, Brazil;; b Department of Biochemistry, Institute of Biology, State University of Campinas (UNICAMP), Campinas, Brazil;; c Department of Plant Biology, Institute of Biology, State University of Campinas (UNICAMP), Campinas, Brazil

**Keywords:** Acylation, lipase, flavonoids, xanthine oxidase

## Abstract

Studies have reported that flavonoids inhibit xanthine oxidase (XO) activity; however, poor solubility and stability in lipophilic media limit their bioavailability and applications. This study evaluated the kinetic parameters of XO inhibition and partition coefficients of flavonoid esters biosynthesised from hesperidin, naringin, and rutin *via* enzymatic acylation with hexanoic, octanoic, decanoic, lauric, and oleic acids catalysed by *Candida antarctica* lipase B (CALB). Quantitative determination by ultra-high performance liquid chromatography–mass spectrometry (UHPLC–MS) showed higher conversion yields (%) for naringin and rutin esters using acyl donors with 8C and 10C. Rutin decanoate had higher partition coefficients (0.95), and naringin octanoate and naringin decanoate showed greater inhibitory effects on XO (IC_50_ of 110.35 and 117.51 μM, respectively). Kinetic analysis showed significant differences (*p* < .05) between the flavonoids before and after acylation regarding *K*_m_ values, whereas the values for *V*_max_ were the same, implying the competitive nature of XO inhibition.

## Introduction

Many *in vitro* and *in vivo* experiments in animals and humans have reported the biological activity of the flavonoids especially that related to their antioxidant action in the prevention and/or combat of chronic degenerative diseases, and as anti-inflammatory and anti-microbial agents and modulators of the activities of enzymes such as xanthine oxidase (XO)^1^.

Xanthine oxidase is an enzyme widely distributed in many species ranging from bacteria to human beings and is present in various mammal tissues^2^. XO catalyses the oxidation of hypoxanthine into xanthine and uric acid during the metabolic processes of purines^3^, producing superoxide radicals (O_2_^−^) and hydrogen peroxide (H_2_O_2_)^4^. Thus, XO is one of the main enzymatic sources of reactive oxygen species (ROS)^5^. Various inflammatory stimuli regulate XO such as lipopolysaccharides, hypoxia, and cytokines and elevated levels of this enzyme can lead to an excessive formation of EROs and consequently oxidative processes that can result in tissue damage^6^.

Some flavonoids have been described as effective XO inhibitors but the low absorption of flavonoids *in vivo* is a limiting factor for their bioavailability^7^^,^^8^. The presence of various hydroxyl groups in the flavonoid molecules endows the compound with some degree of polarity and reduces its lipophilicity^9^^,^^10^. Their low solubility in lipophilic systems also limits the applications of flavonoids in the food, pharmaceutical, and cosmetic industries^7^.

Enzymatic synthesis of acylated derivatives of flavonoids catalysed by lipases has been considered as an effective and promising strategy for improving the liposolubility of these compounds^11^. The great interest in using enzymatic processes to modify molecules is due to their selectivity, especially of the lipases^12^ and the lesser number of stages required in comparison with the classical chemical methods^13^.

Enzymatic acylation of glycosylated flavonoids, catalysed by *Candida antarctica* lipase B (CALB), is highly regioselective; it proceeds exclusively on the primary alcoholic group of the sugar moiety, and the mild conditions of this reaction do not interfere with the flavonoids structures^14^. Some authors have investigated the regioselectivity of CALB in the acylation of rutin and isoquercetin by molecular modelling. According to their reports, the aglycon portion of flavonoids is stabilised at the entrance of the enzyme-binding site by hydrogen bonds and hydrophobic interactions, locating its glycoside residue near the centre of the site. Only the primary 6″-OH of the isoquercetin glucose and the secondary 4″-OH of the rutin rhamnose are acylated, as they stabilise close to the catalytic sites^15^.

Flavonoid selectively acylated with different aliphatic or aromatic acids may not only alter physicochemical properties of these molecules but also improve bioavailability and biological properties compared to the maternal compounds^16^. These include increased capacity to inhibit microsomal lipoperoxidation of isoquercitrin acylated esters^11^, increased antiproliferative activity of long chain acylated esters of quercetin-3-*O*-glucoside^17^, improved antiangiogenic and antitumor properties of silybin^18^, rutin and naringin esters^19^, and inhibition of the sarco/endoplasmic reticulum Ca^2+^-ATPase (SERCA1) by rutin derivatives^20^. On the other hand, the results obtained for the antioxidant capacity of acylated flavonoid derivatives are contradictory. Literature reports found that the acylation of rutin with unsaturated fatty acids, such as oleic, linoleic, and linolenic acid, increased the antioxidant potential of the initial compound^21^^,^^22^. The acylation of isoorientin and isovitexin significantly improved their lipophilicity, but reduced their antiradical activity^23^. This data is in accordance with another report about the antioxidant activity of isoorientin-acylated derivatives, which shows that the derivatives exhibited lower DPPH radical scavenging activity than their parental form isoorientin^24^. A recent study showed that lipophilization of rutin esters (rutin laurate and rutin palmitate) did not improve capacity in bulk oil and in an o/w emulsions compared to untreated rutin^25^.

There has been little work to demonstrate the inhibitory effects of acylated flavonoid derivatives on XO. Acylation of 3-OH group of a new isomer of mesquitol (2,3-*trans*-3′,4′,7,8-tetrahydroxyflavan-3-ol) isolated from *Dichrostachys cinerea* significantly enhanced the α-glucosidase inhibition and displayed xanthine oxidase inhibitory potential^26^. Similarly, acylation of isoquercitrin increased the antioxidant properties, including a higher XO inhibition^27^. Also the acylation of isorhamnetin-3-*O*-glicoside extracted from *Nitraria retusa* using ethyl laurate and ethyl butyrate, catalysed by CALB, increased the XO inhibitory capacity of the compound albeit there was a decrease in radical neutralizing potential^28^. The structural differences, such as the number and position of hydroxyl groups, the nature of saccharidic moieties, as well as the position of glycosidic bonds affect overall conversion yield. For naringin molecule, which possesses primary hydroxyl group on glucose, the acylation takes place on the 6″-OH^21^ since the primary hydroxyl is favoured by CALB. On the other hand, rutin, which has no primary hydroxyl, either the 3″-OH of glucose or the 4″-OH of rhamnose can be acylated^12^^,^^16^^,^^19^.

Our research group previously reported the enzymatic acylation of hesperidin using as acyl donor a medium chain fatty acid (decanoic acid) catalysed by CALB in a two-phase system containing [bmim]BF_4_ and acetone^29^. However, the XO inhibitory capacity of these compounds had not been assessed. Accordingly this study set out to synthesise new acylated derivatives of the flavonoids rutin, naringin, and hesperidin with various monocarboxylic acids (hexanoic, octanoic, decanoic, lauric, and oleic acids) with *Candida antarctica* lipase B (CALB) as the catalyst, in *a bid* to obtain compounds with greater capacity to inhibit XO than the untreated flavonoids. The partition coefficients and the kinetic parameters of the XO inhibition (*V*_max_ and *K*_m_) were assessed for both the untreated flavonoids and their acylated derivatives.

## Materials e methods

### Enzymes and reagents

CALB immobilised in acrylic resin (EC 3.1.1.3, 10,000 U/g) (Novozyme 435), xanthine oxidase from bovine milk, xanthine, allopurinol, hesperidin, naringin, and rutin standards and hexanoic, octanoic, decanoic, lauric, and oleic acids were obtained from the Sigma-Aldrich Chemical Co., St. Louis, MO. All the solvents and reagents were of analytical, spectrometric, or chromatographic quality.

#### Enzymatic synthesis of acylated derivatives of flavonoids with different fatty acids

The enzymatic synthesis of acylated derivatives of flavonoids was performed according to a previously described method^29^^,^^30^ with some modifications. The reaction medium consisted 5 ml of acetone, previously treated with 100 mg/ml 4 Å molecular sieves activated by being kept for 1 h at 100 °C, 12 h prior to the reaction. The molar mass ratio used between the flavonoid and the fatty acid was 1:5 (0.575 mmol of flavonoid and 2.875 mmol of fatty acid). After the solubilisation of the reagents in the reaction medium, 0.65 g of CALB was added for the experiment. The tests were incubated in sealed flasks in a shaker at 45 °C set at 100 rpm and the aliquots were removed after 12 and 24 h of incubation. Purification of the acylated flavonoid derivatives was based on partition between hexane/water (4:1, v/v) system as previously described^31^. After each rinse the mixture was centrifuged (2800 rpm for 2 min) and the organic phase containing the free fatty acids was discarded. The samples were lyophilized, frozen at −20 °C and forwarded for ultra-high performance liquid chromatography–mass spectrometry (UHPLC-MS) analyses to determine the conversion rates of the acylated derivatives.

#### Conversion yields of acylated derivatives of flavonoids determined by UHPLC-MS

The chromatographic separation was achieved using an Acquity UPLC system (Waters, Milford, MA) equipped with a Waters UPLC BEH column (2.1 × 50 mm, particle size 1.7 μm) at a temperature of 30 °C. A 3 μl of each sample was injected and the gradient applied used two mobile phases – (A) ultrapure water with 1% of formic acid and (B) methanol, starting with 5% of B, increasing to 100% B in 8 min, maintained until 8.50 min, and finally returning to the original conditions and stabilising at 10 min. Detection was in negative ions mode using an Acquity TQD electrospray ionization-mass spectrometer (Micromass Waters, Milford, MA) in the following conditions: capillary – 3000 V, cone – 30 V, source temperature 150 °C, and desolvation temperature 350 °C. Quantification of the compounds was achieved using standard curves obtained by injecting standards of the flavonoids in concentrations of ranging from 30 to 300 μg/ml. The conversion yields were calculated from the ratio between the concentration of the acylated derivatives and the initial concentration of flavonoids before the acylation reaction. Calibration curves for each flavonoid were obtained using standards in methanol. The calculations were based on the following equations: hesperidin concentration (μg/ml) = (4 × 10^−5^) (peak area) + 112.28 and rutin/naringin concentration (μg/ml) =  (6 × 10^−5^) (peak area) + 256.71 and the data were expressed in percentage yields (%).

### *Partition coefficient determination in octanol/water (k*)

The partition coefficients of the untreated flavonoids in octanol/water (*k*) were analysed to determine their degrees of lipophilicity. In test tubes, 2.0 ml of a solution of the sample with a concentration of 50 μM were added to 2.0 ml of octanol saturated with water. The mixture was shaken for 1 min and then centrifuged for 15 min at 3000 rpm. After filtering through 0.22 μm polyethylene filter with a PTFE membrane (Merck Millipore, Billerica, MA), the compound concentration was determined for each phase by UHPLC–MS. The partition coefficient was obtained using the equation:

$$k=\frac{\text{Co}}{\text{Ca}\times r};$$

where Co = test compound concentration in octanol, Ca = test compound concentration in aqueous solution, *r* = ratio of the volumes of the oily and the aqueous phases.

### Inhibition of xanthine oxidase (XO) activity by flavonoids and acylated derivatives

Inhibition of XO activity was assessed by measuring the uric acid formed from the xanthine substrate. Solutions of xanthine in various concentrations in a 0.1 M, pH 7.4 phosphate buffer were incubated together with 100 μl of ethanol and the same volume of samples of the solutions with different concentrations (45 and 90 μM in a 0.1 M, pH 7.4 phosphate buffer). The samples were pre-incubated at 37 °C for 10 min. The XO solution (0.3 ml, 0.1 U/ml) was added to the reaction mixture and the flasks were incubated at 37 °C for 20 min. The enzymatic reaction was interrupted by adding 25 μl of 3.2% HCl. The absorption of the samples was measured using an ELISA reader Epoch, BioTek (Winooski, VT) at 290 nm. The uric acid production was calculated from the differential absorbance with a blank solution without xanthine oxidase. The control was a solution containing xanthine and XO. Allopurinol, a specific XO inhibitor, was used as a positive control. XO inhibitory activity was expressed as the percentage inhibition of XO (XOI, %) by using the following equation:

$$XOI\;\left(\%\right)=\left(1-\frac{\text{Abs}\text{am}\;}{Absc}\right)\times 100;$$

where Absc and Absam = absorbance values for the control reaction and for the test samples, respectively.

### Measurement of the kinetic constants

The enzymatic kinetics trials were made with those compounds that showed the highest percentage inhibition (XOI) of XO activity. *V*_max_ (maximum reaction rate) and *K*_m_ (Michaelis–Menten constant, that is the substrate concentration at 1/2 the maximum reaction rate) were calculated and the type of inhibition kinetics was identified using the Origin 8.6 program and the rectangular hyperbola model. The Michaelis–Menten equation linearized by Lineweaver–Burk was used to determine *V*_max_ and *K*_m_ by plotting a graph, that is 1/*V* against 1/[substrate concentration], and estimated by the intercept and slope respectively. Reaction rate (expressed as uric acid concentration in μmol/min) was obtained from the uric acid standard curve (*y* = 0.024*x* − 0.1242, *R*^2^ = 0.9786). Those concentrations associated to 50% inhibition (IC_50_) were also calculated.

### Statistical analysis

The experiments were performed in triplicate ANOVA one way was used to analyse statistical significance followed by Turkey (Bonferroni) test using Origin Pro 8 (OriginLab, Northampton, MA) statistics software. The results are presented as mean ± SD and statistics were considered significant when the *p* values was .05 or less.

## Results and discussion

### Enzymatic synthesis of the acylated derivatives of flavonoids

Table 1 shows the conversion yields derivatives of naringin, hesperidin, and rutin acylated using hexanoic, octanoic, decanoic, lauric, and oleic acids.

**Table 1.** Conversion yields (%) of acylated flavonoid derivatives with monocarboxylic acids (hexanoic, octanoic, decanoic, lauric, and oleic acids).

Untreated flavonoids	Flavonoid acylated derivatives	Time (h)	Conversion yields (%)
Naringin	Naringin hexanoate	12	27.30
		24	13.66
	Naringin octanoate	12	38.40
		24	28.89
	Naringin decanoate	12	9.22
		24	24.32
	Naringin laurate	12	–
		24	–
	Naringin oleate	12	–
		24	–
Hesperidin	Hesperidin hexanoate	12	33.00
		24	31.00
	Hesperidin octanoate	12	44.00
		24	44.70
	Hesperidin decanoate	12	23.50
		24	24.60
	Hesperidin laurate	12	32.40
		24	41.90
	Hesperidin oleate	12	31.80
		24	64.20
Rutin	Rutin hexanoate	12	24.90
		24	20.27
	Rutin octanoate	12	37.95
		24	51.29
	Rutin decanoate	12	34.65
		24	28.71
	Rutin laurate	12	–
		24	9.34
	Rutin oleate	12	–
		24	1.53

The highest conversion yields were obtained for hesperidin, followed by rutin and naringin. Acylation of rutin and naringin was more favourable with the shorter chain hexanoic (6C), octanoic (8C), and decanoic (10C) fatty acids. Lauric (12C) and oleic (18C) acids are poor substrates for the acylation of rutin and naringin albeit they efficiently esterified the hesperidin molecule. Earlier studies have reported that the highest CALB-catalysed biosynthesis yields of acylated derivatives of naringin and rutin are obtained using fatty acids with up to 10C^21^^,^^32^. Any further increase of the number of C in the fatty acid chain seems to impede esterification and reduce the efficiency of enzymatic conversion^33^. According to a recent study, isoquercitrin (quercetin-3-*O*-*β*-D-glucopyranoside) can be efficiently substituted at 6″-OH by acetate or by C4- to C16-aliphatic acids by CALB. Shorter dicarboxylic acids (C2 to C4) were not substrates for the lipase and did not react at all, while the enzyme has accepted C5- to C12-dicarboxylic acid^11^.

### Kinetics of xanthine oxidase (XO) inhibition

**Figure 1.**
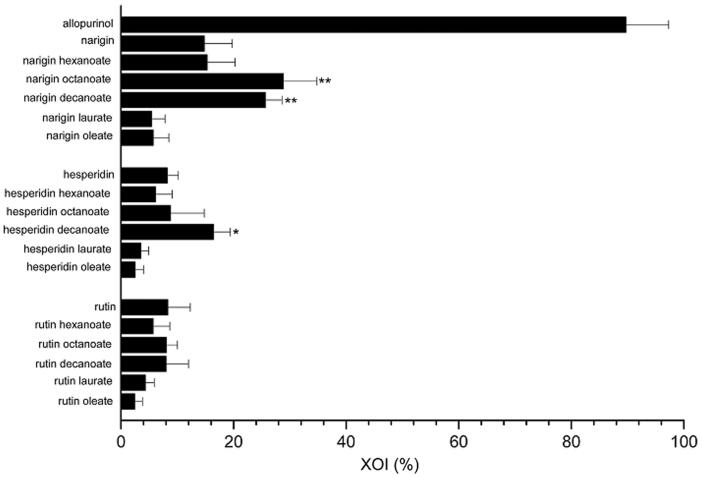
Xanthine oxidase inhibition (XOI %) by different untreated flavonoids and their acylated derivatives at 45 μM using 250 μM of xanthine substrate. *p < .05, **p < .01 compared with similar untreated flavonoid.

Figure 1 displays the percentages of XO inhibition (XOI) obtained for the respective compounds before and after 12 h of enzymatic acylation.

There was a considerable increase in the inhibitory effect on XO activity associated to three of the acylated derivatives, naringin octanoate, naringin decanoate, and hesperidin decanoate (28.80 ± 5.76, 25.61 ± 2.85, and 16.42 ± 3.21, respectively), compared to the untreated flavonoids (14.78 ± 4.80 and 8.24 ± 2.10 for naringin and hesperidin, respectively). Compared to allopurinol, which strongly inhibited XO (89.67%), the derivatives biosynthesised with naringin and hesperidin showed a moderate, but rather promising, activity. For the rutin derivatives, however, the acylation reaction did not enhance the inhibition of XO activity. In the light of those results, the three acylated compounds were selected to assess the kinetic parameters involved in XO inhibition (*V*_max_, *K*_m_, and IC_50_) and to determine their partition coefficients in octanol/water (*k*).

**Figure 2.**
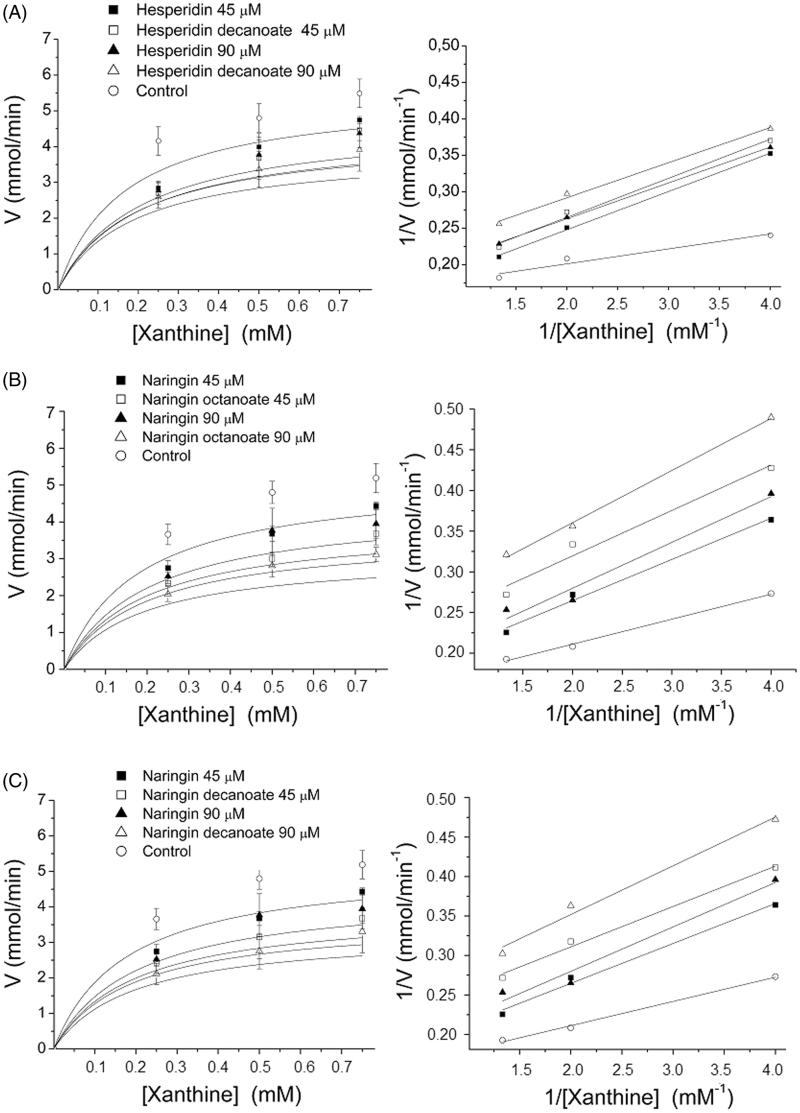
XO inhibition kinetics using Michaelis–Menten curve and Lineweaver–Burk double reciprocal plots for hesperidin decanoate (A), naringin octanoate (B), and naringin decanoate (C) at 45 and 90 μM.

Two concentrations (45 and 90 μM) of the inhibitors hesperidin decanoate, naringin octanoate, and naringin decanoate and three concentrations of xanthine (250, 500, and 750 μM) were used to determine the kinetic parameters. The reactions were performed in the presence and absence of the inhibitor to obtain the Michaelis–Menten curves and the Lineweaver–Burk double reciprocal plots as shown in Figure 2.

The response of the acylated flavonoids naringin octanoate, naringin decanoate, and hesperidin decanoate in inhibiting XO activity is directly proportional to their concentrations and 90 μM is the concentration with the greatest effect in reducing the reaction rate (uric acid concentration formed/minute). The results were analysed by means of the Lineweaver–Burk double reciprocal plots and the *K*_m_ and *V*_max_ values obtained (Table 2) by graphic extrapolation. The *V*_max_/*K*_m_ ratio was also calculated to determine catalytic efficiency. The double reciprocal graph (*B*), expressed as 1/*V*_o_ (*y*) plotted against 1/[*S*] (*x*) produces a straight line for which the slope gives the value of *K*_m_/*V*_max_, the intercept on the 1/*V*_o_ axis is equal to 1/*V*_max_ and the intercept on the 1/[*S*] axis is equal to −1/*K*_m_.

**Table 2.** Kinetic parameters of the enzymatic reaction catalysed by xanthine oxidase in the absence (control) and presence of the inhibitors (45 and 90 μM).

Inhibitor	[Inhibitor] (μM)	*V*_max_ (mmol/min)	*K*_m_ (mM)^a^	*V*_max_/*K*_m_
Control	0	5.19 ± 0.15	0.17	30.52
Naringin	45	4.43 ± 0.07	0.20**	22.15
	90	3.68 ± 0.03	0.18	20.44
Naringin octanoate	45	3.94 ± 0.91	0.19*	20.74
	90	3.11 ± 0.18	0.17	18.29
Naringin decanoate	45	3.94 ± 0.63	0.19*	20.74
	90	3.30 ± 0.70	0.20**	16.50
Hesperidin	45	4.74 ± 0.67	0.20**	23.70
	90	4.37 ± 0.27	0.19**	23.00
Hesperidin decanoate	45	4.47 ± 0.45	0.20**	22.35
	90	3.91 ± 0.78	0.22**	17.77

* *p* < .01.

** *p* < .001 compared with control (without inhibitor).

a SD <0.002.

Given that the *V*_max_ values show no significant alterations and that the *K*_m_ (called the apparent *K*_m_ and expressed as the [*S*] in which *V*_o_ = ½*V*_max_) were higher in the presence of inhibitors when compared to the control, then it can be supposed that there is a competitive, reversible inhibition reaction mechanism of the untreated and acylated flavonoids. The competitive inhibitors are those most similar to the substrate and they, therefore, occupy the active site. The occupation by the inhibitor thus prevents the substrate from connecting to the active site of the enzyme. Whenever [*S*] greatly exceeds [*I*], the probability of an inhibitory molecule connecting to the enzyme is minimized and the reaction will show a normal *V*_max_ value. That effect on the increase in the apparent *K*_m_, together with the absence of any effect on the *V*_max_, is the diagnosis for competitive inhibition and it is readily revealed in the double reciprocal plots. To compare the inhibitory efficiency of the compounds in regard to XO, the *V*_max_/*K*_m_ ratios provide better evidence. As seen in the results, the *V*_max_/*K*_m_ ratios were higher when acylated flavonoid derivatives were used as inhibitors compared to similar untreated flavonoids.

That finding is in agreement with an earlier study that evaluated the inhibitory effect of apigenin, quercetin, myricetin, genistein, and isovitexin on XO activity and reported that they all performed as competitive inhibitors^34^. The presence of sugar units in flavonoid structures also reduces their inhibitory power so that an aglycone form such as quercetin is a more efficient inhibitor than the glycosylated form, rutin^34^^,^^35^.

**Figure 3.**
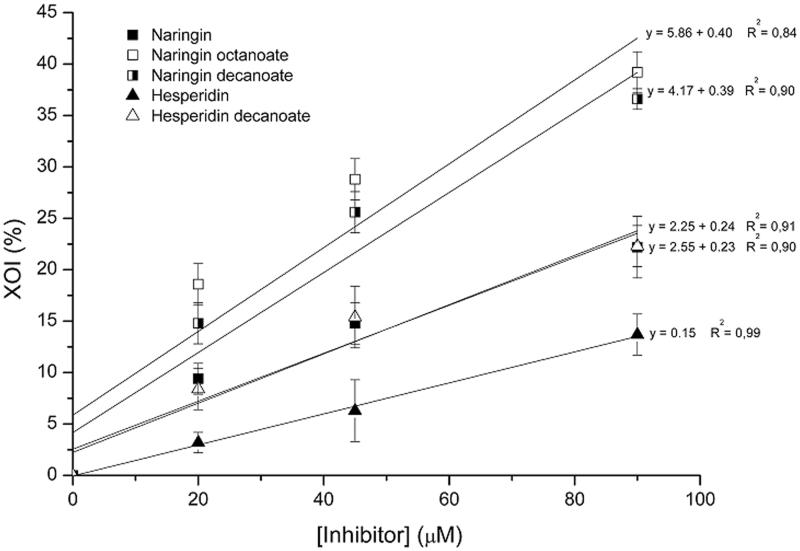
Effect of concentrations of untreated flavonoids and acylated derivatives on xanthine oxidase inhibition (XOI %).

The XOI (%) as a function of flavonoid concentration and the IC_50_ values are displayed in Table 3 and Figure 3. For each equation corresponding to the straight lines of the graph, calculations were made to obtain the value of *x* when *y* = 50 (IC_50_). It can be seen that the acylation reaction led to a reduction in the IC_50_ for the three acylated derivatives. Naringin octanoate showed the greatest inhibitory effect on XO activity (IC_50_ = 110.35 μM), followed by naringin decanoate (IC_50_ = 117.51 μM) and hesperidin decanoate (IC_50_ = 198.96 μM), whereas the standard inhibitor of xanthine oxidase (allopurinol) had an IC_50_value of 14.67 μM.

**Table 3.** IC_50_ (μM) of untreated flavonoids and acylated derivatives.

Inhibitor	Straight line equation	*R*^2^ value	IC_50_ (μM)
Naringin	*y* = 2.55 + 0.23*x*	0.90	206.30
Naringin octanoate	*y* = 5.86 + 0.40*x*	0.84	110.35
Naringin decanoate	*y* = 4.17 + 0.39*x*	0.89	117.51
Hesperidin	*y* = 0.15*x*	0.99	333.33
Hesperidin decanoate	*y* = 2.25 + 0.24*x*	0.91	198.96
Allopurinol	–	–	14.67

Higher inhibition activities were reported for isoquercitrin oleate (C18:1), which presents an unsaturation in the acyl group, when compared to its saturated analogue (IC_50_ values of 27 and 61 μM, respectively)^28^. The authors observed an increase in the IC_50_ values of saturated esters with the decrease in the carbon chain length (from 61 to 144 μM for isoquercitrin stearate and butyrate, respectively)^28^.

Based on the structure of flavonoids, a method for predicting IC_50_ values of XO by calculating the contribution of each hydroxyl moiety towards inhibition of this enzyme was described^36^. The group with the strongest negative contribution to inhibition of XO is the 2′-hydroxyl moiety as can be concluded from comparing kaempferol with morin in which the IC_50_ increases from 2.5 to 40 μM, respectively. The flavones (lutein and apigenin) and the flavonols (like quercetin, kaempferol, and miricetin) are capable of inhibiting the activity of this enzyme even when they are only present in low concentrations^36–38^. Hydroxyl groups at C-5 and C-7 and the double bond between C-2 and C-3 were described as essential for high inhibitory activity against XO^39^. The planar structure of flavones and flavonols and the C2 = C3 double bonds of flavonoids were considered advantageous for XO inhibition^34^. Unfortunately those authors did not assess hesperidin, naringin, or any other acylated flavonoid derivative.

### Partition coefficients of acylated derivatives of flavonoids in 1-octanol/water

Partition coefficient in 1-octanol/water (*k*) was measured for all prepared compounds as a basic empirical determination of hydro- or lipophilicity (Table 4). This value is a parameter that relates single-solute partitions between polar (water) and nonpolar (octanol) phases, which determines *in vitro* solubility in appropriate pharmaceutical and cosmetic preparations.

**Table 4.** Partition coefficient in octan-1-ol/water (*k*) of untreated flavonoids and their acylated derivatives.

Compound	Partition coefficient (*k*)^a^
Quercetin	1.46
Naringin	0.37
Rutin	0.48
Hesperidin	0.42
Naringin octanoate	0.86
Naringin decanoate	0.86
Rutin decanoate	0.95
Hesperidin decanoate	0.83

a SD <0.01.

Among the flavonoids and their acylated derivatives that have been analysed, the aglycone form, quercetin, is the one that presents the highest partition coefficient and therefore the highest degree of lipophilicity (1.46). That high partition coefficient of quercetin in an octanol/water system when compared to its glycosylated form has already been reported^39^^,^^40^. The derivatives biosynthesised with decanoic acid had partition coefficients of 0.95, 0.83, and 0.86 for rutin decanoate, hesperidin decanoate, and naringin decanoate, respectively. Those values were higher than the coefficients obtained for the untreated flavonoids, 0.48, 0.42, and 0.37 for rutin, hesperidin, and naringin, respectively. The same *K* values were obtained for the naringin derivatives whether they were acylated with 8C chains or 10C chains.

The variety of substituents on the flavonoid molecules largely influence their physicochemical properties such as dipole moment or hydrophobicity, and thus determine the partitioning into lipid membranes^41^. Flavonoid acylated derivatives are expected to exhibit a higher affinity for phospholipidic membranes and so to be transferred into cells^28^. The result indicated that the degree of lipophilicity played a major role in improving enzyme inhibitory activity. These results are in alignment with reports in the literature which indicate that increasing the lipophilicity of the flavonoid molecule enhances its inhibitory effect on XO, as has been described for isoquercitrin^27^, insofar as it increases the accessibility of the compound to the active site of the enzyme^26^.

## Conclusion

Flavonoid acylation, as has been described, provides a useful tool for flavonoid ester formation with improved characteristics. The results suggest that selective enzymatic synthesis of acylated flavanone derivatives catalysed by CALB lipase may represent a new approach to the production of XO competitive inhibitors with greater lipophilicity. The most potent XO inhibition was observed in naringin derivatives with octanoic and decanoic acids (naringin octanoate and naringin decanoate). The results provide the basis of the kinetics of the interaction mechanisms of acylated flavonoid derivatives with XO that may lead to the development of potential new drugs for XO inhibition. This approach also enables the use of flavonoid fatty acid esters in oil-based systems. Thus, these acylated derivatives are promising candidates to be used in pharmaceutical, cosmetic, and nutritional preparations for preventive and/or therapeutic purposes.
